# Effectiveness of family-centered intervention programs on objectively measured physical activity in children aged under 13: a meta-analysis of randomized controlled trials

**DOI:** 10.3389/fpubh.2025.1611496

**Published:** 2025-07-15

**Authors:** Qian Wang, Lawrence T. Lam, Heng Lin, Wenxian Yang, Fuxian Yin, Yongwei Li

**Affiliations:** ^1^Macau University of Science and Technology, Taipa, Macao SAR, China; ^2^Jiangsu Sports Science Research Institute, Nanjing, Jiangsu, China

**Keywords:** family intervention, physical activity, exercise, parent, child

## Abstract

**Introduction:**

This review aims to evaluate the efficacy of family-centered physical activity interventions, as assessed through randomized controlled trials (RCTs) on objectively measured moderate-to-vigorous physical activity (MVPA) and sedentary behavior (SB) in children under 13. To ensure higher quality and reduce measurement bias, a quantitative approach was employed.

**Methods:**

A detailed search was systematically conducted in PubMed, Medline, Web of Science, and Embase for studies published between January 2013 and February 2024. Only RCTs investigating the efficacy of family-centered interventions using objective measurements in children under 13 were included. Study characteristics were systematically summarized, and the risk of bias was assessed using the Cochrane risk of bias tool. Meta-analyses were performed to evaluate the effectiveness of interventions, and subgroup analyses were conducted in RevMan 5.4 to explore potential effects.

**Results:**

Ten studies, comprising a total of 1,557 parent-child dyads, met the inclusion criteria. The mean age of participants ranged from 3 to 11 years. The studies assessed various outcomes, including MVPA and sedentary time. Meta-analysis revealed that family-centered interventions were significantly associated with increased MVPA (WMD = 5.13, 95% CI = 1.09 to 9.17, *p* = 0.01). However, no significant difference in SB was found between the intervention and control groups (WMD = −2.24, 95% CI = −9.33 to 4.86, *p* = 0.54). Subgroup analyses showed significant effects for short-term interventions (WMD = 9.08, 95% CI = 2.54 to 15.62, *p* = 0.007) and on weekends (SMD = 0.63, 95% CI = 0.33 to 0.93, *p* < 0.05).

**Conclusions:**

Family-centered interventions are a promising approach to enhancing children's MVPA, particularly in the short-term and on weekends. However, the effect on reducing SB appears limited. Future research should focus on larger, more diverse samples (e.g., populations in developing countries), utilize high-quality measurement tools, and novel outcomes (e.g., FMS) to better assess the effectiveness of these interventions.

**Trial registration:**

Meta-analysis PROSPERO: CRD42023488011.

## 1 Introduction

Physical activity (PA) plays a crucial role in the growth of children. However, according to the WHO's *Global Status Report on Physical Activity 2022*, a significant 81% of adolescents worldwide fail to meet the PA thresholds recommended by the organization (1). PA is defined as any bodily movement produced by skeletal muscles which leads to energy expenditure (2). It is typically categorized into three levels: light, moderate, and vigorous (3). Numerous studies have shown that regular moderate-to-vigorous physical activity (MVPA) is beneficial for both physical and mental health (4). The WHO recommends at least 60 min of MVPA per day for children (5–7). Insufficient PA has become a public health crisis globally (8). PA encompasses any bodily any bodily movement that contributes to the improvement or maintenance of physical fitness, health, and overall wellbeing (9). Evidence indicates that physical inactivity in the process of childhood is harmful to both physical and mental health, increasing the risk of some chronic diseases (10), such as obesity (11, 12), type 2 diabetes (13), cardiovascular diseases (14), and cancer (15). Consequently, health promotion efforts should prioritize increasing physical activity levels, especially among children.

Based on social cognitive theory, inactive children are closely influenced by their parents (16). This theory, widely applied across various fields such as business (17), media (18), and healthcare (19), asserts that human behavior is influenced by the dynamic interaction of three fields, like personal, behavioral, and environmental factors (20). People are not only products of their environments, but also actively shape their environments. Behavior regulation is thus a dynamic process (21), involving a continuous interaction between personal, behavioral, and environmental elements (20). Social cognitive theory emphasizes that knowledge acquisition and learning occur through the observation of models (22). Within the family system, parental involvement is crucial for child development (23). Parent–child dyads, embedded within a network of interdependencies, function collectively to promote shared growth, and mutual understanding (24). When they engage in PA together, both benefit from the enriched family environment, creating a win-win situation (25). Family-centered interventions, therefore, could become the potential chance to significantly promote children's PA (26, 27). Over the past two decades, research on family-based interventions has evolved in both study design and methodology. Early studies primarily employed observational designs (28), while recent research has increasingly utilized RCTs (29, 30), offering more robust designs. Although challenges such as participant engagement and adherence persist, new issues have emerged, including the integration of digital tools into interventions (31, 32). Furthermore, there has been a shift in research methodologies, with earlier studies relying heavily on self-reported data (33, 34), while more recent studies incorporate objective measurements (e.g., wearables) (35, 36).

Still, there remains a gap in assessing the overall effectiveness of such family-centered interventions. Some researchers argue that conclusions on the efficacy of family-centered interventions remain unclear (23, 37). Some studies have shown that family-centered intervention have a great significance on improving physical activity (25, 26, 38). While certain studies suggest significant improvements in physical activity, the design of family-centered interventions may not always be convincing. In accordance with the 2011 Levels of Evidence, evidence is hierarchically classified into four distinct levels, ordered from highest to lowest: RCTs, cohort and case-control studies, case series, and at the lowest level, expert opinions that lack explicit critical evaluation or systematic appraisal (39). Therefore, only high-quality articles, specifically RCTs, should be included in this review.

Moreover, some researchers have investigated the efficacy of family-centered PA interventions using both objective and subjective measurement devices, which might introduce a high degree of measurement bias. In the context of PA, “skeletal muscles” and “energy expenditure” refer to specific mechanistic actions (2). The amount of energy expended during an activity which can be quantified in kilojoules or kilocalories. There are notable differences in how energy expenditure is measured, including subjective methods like self-reporting and objective devices like the Actigraph accelerometer (40). There are different in the amount of energy of measurement in the self-reporting (9). Various questionnaires, such as International Physical Activity Questionnaire (IPAQ), are mostly used to assess the type and time of PA in our daily life (41). While IPAQ is widely used due to its feasibility and cost-effectiveness, its validity has not been thoroughly evaluated. On one hand, evidence supports that IPAQ demonstrates reasonable measurement properties across diverse settings, including monitoring the levels of PA in adults who aged from 18 to 65 across 12 countries (42). On the other hand, Studies have indicated that participants who self-report PA using the IPAQ-SF generally report higher levels of vigorous physical activity and lower amounts of sedentary time compared to data derived from accelerometer-based measurements (43). Additionally, subjective measurements, like those from self-report questionnaires, may not be suitable for younger children. A study indicated that for children aged under 14 years in Europe, the validity of self-report methods was poor, and objective methods, such as the ActiGraph accelerometer, may be a more applicable alternative (44). In summary, there are clear differences between self-report questionnaires and objective measurements in terms of reliability and validity, with objective measures like the ActiGraph accelerometer being more suitable for younger children.

This review improves upon previous studies by including high-quality articles and minimizing measurement bias. The aim is to investigate the effectiveness of family-centered RCT physical activity intervention programs, which use objective devices to measure outcomes in children under 13, through a quantitative approach.

## 2 Method

This article fully complies with the PRISMA (Preferred Reporting Items for Systematic Reviews and Meta-Analyses) guideline (45), and adheres to the PRISMA checklist to maintain research quality, covering aspects such as search strategy, and data extraction.

### 2.1 Information source

A comprehensive systematic search of four scientific medicine literature databases was conducted to access the most recent papers, covering the period from January 2013 to February 2024. The databases included Web of Science, Embase, Medline, and PubMed.

### 2.2 Search strategies

A total of 6233 articles were retrieved using search terms based on the PICO principle in the four databases (Appendix 1). The search terms were combined by adding “AND” for different concepts and adding “OR” for similar terms. Then all articles were subsequently imported into the Endnote X9 for reference management.

### 2.3 Inclusion criteria

The review of inclusion criteria is based on the PICO framework, and give a further explanation in the study type and sample. (1) Participants: Children under 13 years of age, or studies with a mean age under 13, and parent-child dyads (where at least one parent and one child are directly involved in the physical activity together). There is no restriction on the number of individuals in the family (e.g., father/mother-son/daughter). (2) Interventions: Any family-centered physical activity interventions where both the parent and child engage together. There are no restrictions on the intervention setting (e.g., laboratory, classroom, home, hospital, gym) or duration. The FITTVP components of physical activity (i.e., frequency, intensity, time, type, volume, progression) are not limited. (3) Comparison: Studies must include a comparison condition (e.g., intervention group vs. control group, waitlist group, or usual care group). (4) Outcomes: The study must report on objective measures of: MVPA time (e.g., minutes per day). Sedentary behavior (e.g., sedentary time in minutes per day). Daily steps (e.g., steps per day). (5) Study Type: Only RCTs are included. (6) Study Samples: There are no restrictions on the sample size (e.g., pilot RCTs with limited sample sizes are included).

### 2.4 Exclusion criteria

(1) Non-English Literature: Studies published in languages other than English. (2) Literature Reviews and Non-Original Research: literature reviews, patent literature, protocols, and studies with missing physical activity data. (3) Health Conditions: Studies where participants have physical diseases or exercise-related disorders. (4) Duplicate Samples: Articles using the same sample or dataset. (5) Non-Objective Measurements: Studies where children's physical activity levels are not objectively measured. (6) Unclear or non-family-centered Interventions: Studies with interventions that do not specifically involve family-centered physical activity.

### 2.5 Data extraction and management

Two authors (WQ and LH) and a qualified research assistant independently screened all articles and extracted the data. Any conflicts were addressed through discussion until unanimous agreement was achieved. Data were extracted according to the following aspects: (1) study characteristics (i.e., title, authors, publication year), (2) participant characteristics (i.e., age, sample size), (3) study design (i.e., two-arm RCT, three-arm RCT, cluster, stratified), (4) intervention duration, (5) intervention programs, and (6) the mean and standard deviation values of outcomes. If multiple studies used the same data, they were combined into one for analysis. In cases of missing data, the authors contacted the original researchers to obtain the necessary information. Data privacy and participant protection are critical ethical concerns in contemporary research. While advocating for data openness, it is essential to implement both technical and ethical measures to ensure the adequate protection of participants' privacy (46).

### 2.6 Quality of assessment

The risk of bias in the included studies was independently evaluated by two reviewers (YWX and LYW) utilizing the Cochrane Risk of Bias Tool (version 2.0). Discrepancies in the assessments were resolved through a deliberative process involving a third expert to reach a consensus. The tool assesses seven domains of bias risk. Each domain was classified as having a low, high, or unclear risk of bias. The evaluation of study quality was subsequently conducted based on the ratings within these domains. The resultant risk of bias chart was generated using RevMan 5.4 software to facilitate data presentation and analysis.

### 2.7 Statistical analysis

The review used a random-effects model to assess the degree of heterogeneity across the included studies. RevMan 5.4 was used to perform the primary analyses, including generating forest plots, conducting heterogeneity tests, performing sensitivity analysis, and carrying out subgroup analysis. For studies with different units of measurement, Statistical analysis was undertaken employing the standard mean difference (SMD) as the effect size measure, with a 95% confidence interval (95% CI) to provide an estimation of the precision and uncertainty associated with the calculated effect size (47). For studies with the same outcome variable and measurement unit, the weighted mean difference (WMD) was calculated. A *p*-value of < 0.05 was considered statistically significant for the differences between groups. Based on the characteristics of the selected studies, subgroup analysis was performed according to the primary outcome, intervention content, and duration (short-term, medium-term, or long-term) to evaluate whether the effect sizes differed across these subgroups.

The heterogeneity across the included studies was quantified using the *I*^2^ statistic, with *I*^2^ values classified as very low ( ≤ 25%), low (25% < *I*^2^ ≤ 50%), moderate (50% < *I*^2^ ≤ 75%), and high (>75%). To assess potential publication bias, Egger's test was applied. Additionally, a sensitivity analysis was performed to examine the robustness of the findings by systematically excluding each study in turn, thereby assessing whether the removal of any single study had a substantial impact on the pooled effect size.

## 3 Results

### 3.1 Study selection

**Figure 1:**
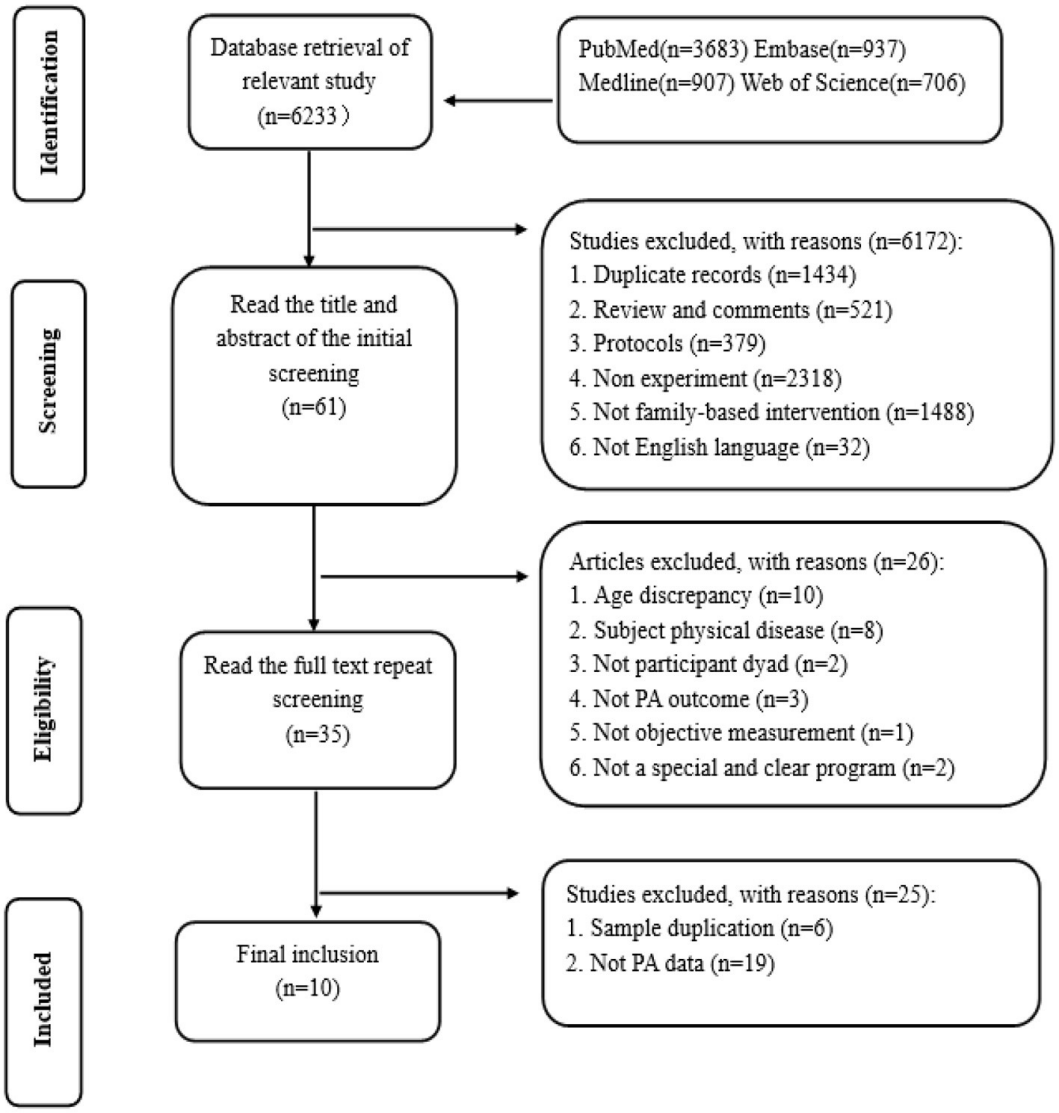
Article selection flow chart for the meta-analysis.

A comprehensive search across four medical databases yielded a total of 6,233 studies by using predefined search terms. After applying the established inclusion and exclusion criteria, 6,222 studies were excluded. Ultimately, 10 studies were selected for inclusion in the systematic and quantitative analysis, as depicted in Figure 1.

### 3.2 Study characteristics

This review includes 10 RCTs published between 2013 and 2024, comprising a total of 1,557 parent-child dyads involved in family-centered PA interventions (Table 1) (29, 30, 35, 36, 48–53). The participants' mean age ranged from 3 to 11 years, with the largest study including 826 parent-child dyads (53), and the smallest involving (50). All studies adhered to RCT methodology, all studies were the randomized controlled trial, with two being pilot RCTs (35, 50), three cluster RCTs (one of which was a cluster randomized clinical trial) (29, 30, 53), and one crossover RCT (48). Among the selected studies, three were published after 2020 (29, 30, 49). Three studies were from USA (49, 50, 52), and two studies were from Australia (35, 48). The left studies, each of one, came from China (30), Canada (36), Danish (29), UK (51), German (53), respectively. Of the 10 studies, nine involved both child-parent dyads, while one study focused only on child-father dyads. Five of the selected studies used the same devices, the Actigraph GT3X^+^. Two articles used the GT1M Actigraph accelerometer (50, 51). The rest of objective devices were Atciheart (53), Actical accelerometer (48), and non-commercial DeviceTracker apps (29). Theories used in the selected studies included socio-ecological theory (52), social cognitive theory (30, 49, 50), social learning theory (53), self-determination theory (51), theory of planned behavior (35, 48), family system theory (30), planning and self-regulation theory (36), and the behavioral model Social Cognitive Theory (29). The social cognitive theory was the most commonly applied framework in these family-centered interventions. The duration of interventions varied widely, ranging from 2 weeks to 2 years (29, 52). Each intervention program had a clear focus, such as teamplay (51) and mHealth intervention (49) and so on. The outcomes of the interventions were diverse, with many studies evaluating the MVPA of both children and parents separately, alongside other indicators such as sedentary behavior and daily step count.

**Table 1:** Characteristics results of a meta-analysis on the family-centered physical activity intervention.

**Publication**	**Country**	**Monitoring device**	**Participant (dyads)**	**Age (mean ±SD)**	**Theory**	**Program**	**Duration**	**Outcome**	**Study**
De Bock et al. (53)	German	Atciheart device	826	5.0 ± 0.2	Social learning	Participatory intervention	12 months	MVPA, SB	Two-armed cluster RCT
Jago et al. (51)	UK	GT1M Actigraph accelerometer	75	7.3 ± 3.5	Self-determination theory	Teamplay intervention	8 weeks	MVPA	Two-armed RCT
Straker et al. (48)	Australia	Actical accelerometer	56	11.3 ± 0.8	NA	The replacement of electronic games at home intervention	8 weeks	MVPA, SB	Crossover RCT
Jake-Schoffman et al. (50)	USA	GT1M Actigraph accelerometer	33	11 ± 0.6	Social cognitive theory, the theory of planned behavior, family systems theory	Motivated interactive technology with family intervention	12 weeks	MVPA, Steps	Two-armed pilot RCT
French et al. (52)	USA	Actigraph GT3X +	534	3.4 ± 0.7	Social ecological theory	Now Everybody Together for Amazing and Healthful Kids (NET-Works) intervention	3 years	MVPA, SB	Two-armed RCT
Yoong et al. (35)	Australia	Actigraph GT3X +	76	4.4 ± 0.5	The theory of planned behavior	Sleep intervention	3 months	MVPA, PA	Two-armed pilot RCT
Rhodes et al. (36)	Canada	Actigraph GT3X +	102	8.9 ± 2.1	Planning and self-regulation theory	Parental planning skills intervention	26 weeks	MVPA	Two-armed RCT
Pedersen et al. (29)	Danish	Non-commercial DeviceTracker apps	89	9.1 ± 2.6	The behavioral model social cognitive theory	Screen media reduction intervention	2 weeks	MVPA, leisure nonsedentary	Two-armed cluster randomized clinical trial
Staiano et al. (49)	USA	Actigraph GT3X +	72	4.0 ± 0.8	Social cognitive theory	mHealth intervention	12 weeks	MVPA, SB,	Two-armed RCT
He et al. (30)	China	Actigraph GT3X +	108	4.5 ± 0.6	Social cognitive theory	Family-based parent-led intervention	8 weeks	MVPA	Two-armed cluster RCT

### 3.3 Risk of bias

**Figure 2:**
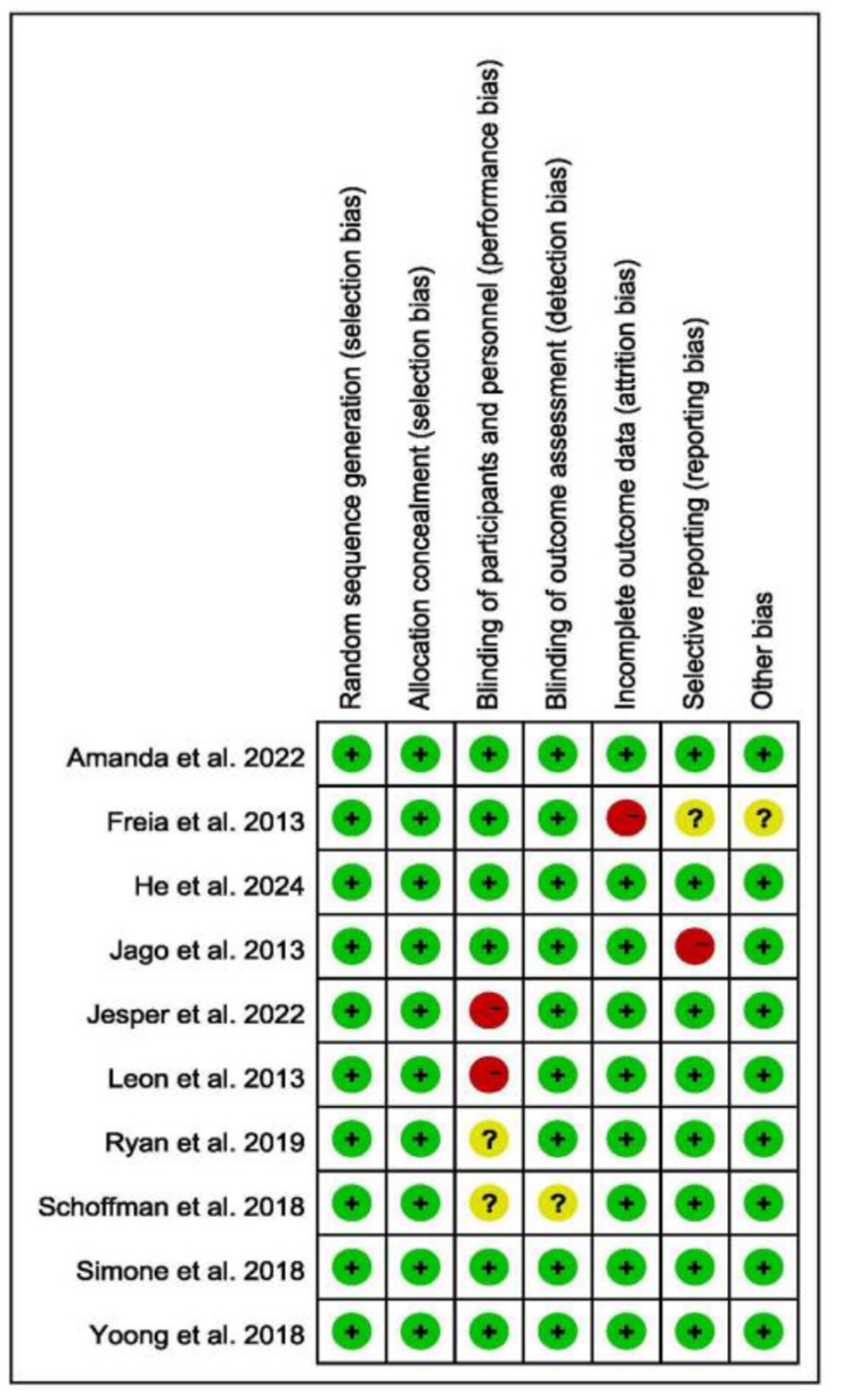
Risk of bias graph each risk of each article.

**Figure 3:**
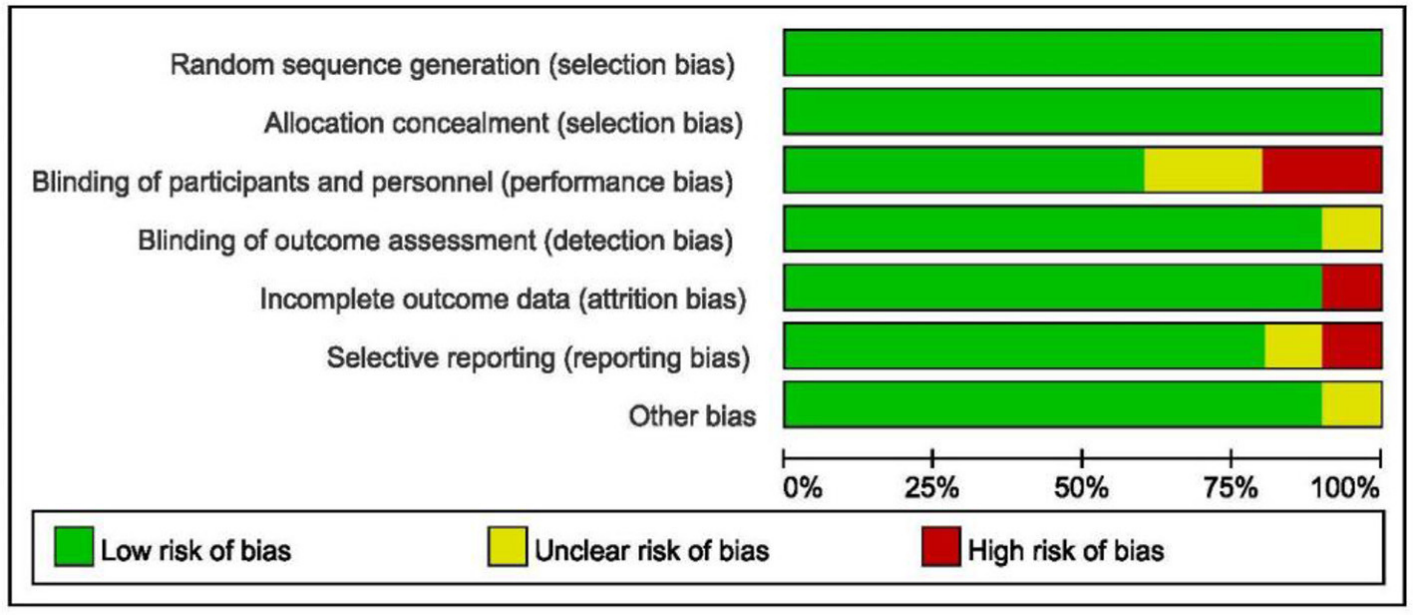
Risk of bias graph each risk of bias item presented as percentages.

Due to the RCT design, all studies involving randomization exhibited low selection bias (Figure 2). Additionally, most of the selected studies provided a clear flow of participant enrollment and demonstrated low attrition bias. The included studies adhered to methodological standards, including allocation concealment and blinding of participants and personnel. Importantly, since outcomes were measured using objective monitoring devices, detection bias was minimal. The researchers also focused on other potential biases, as shown below (Figure 3).

### 3.4 Results of meta-analysis of family-centered interventions

#### 3.4.1 The results of MVPA

**Figure 4:**
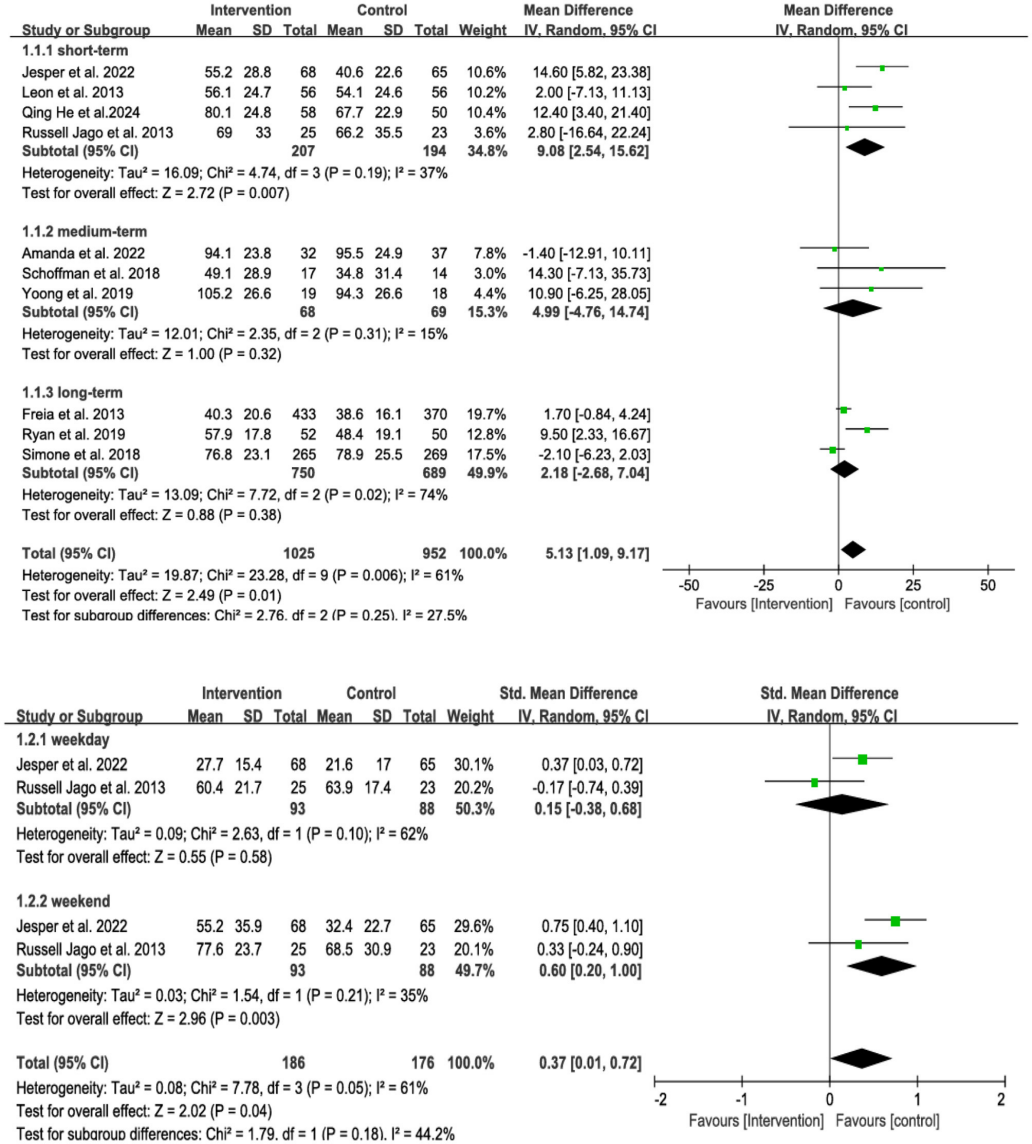
Subgroup of the forest plot of family-centered intervention on MVPA in children aged under 13.

Family-centered interventions were significantly associated with an increase in children's MVPA (mean min/day) compared to controls (WMD = 5.13, 95% CI = 1.09, 9.17, *p* = 0.01, *I*^2^ = 61%, *p* = 0.006). To explore the sources of high heterogeneity, a subgroup analysis was conducted based on intervention duration (short-term: ≤ 8 weeks; medium-term: >8 weeks and ≤ 16 weeks; long-term: >16 weeks) and time of intervention (weekday vs. weekend) (Figure 4). A significant difference was found in the duration subgroup (*p*=0.03). Specifically, a significant effect was observed in the short-term group (WMD = 9.08, 95% CI = 2.54 to 15.62, *p* = 0.007), with low heterogeneity (*I*^2^ = 37%, *p* = 0.19). In contrast, the medium-term (WMD = 4.99, 95% CI = −4.76 to 14.74, *p* = 0.32) and long-term groups (WMD = 2.18, 95% CI = −2.68 to 7.04, *p* = 0.38) did not show significant effects. There was also a significant difference between weekdays and weekends (*p* < 0.05), with a notable effect on weekends (SMD = 0.63, 95% CI = 0.33 to 0.93, *p* < 0.05) and low heterogeneity (*I*^2^ = 35%, *p* = 0.21).

**Figure 5:**
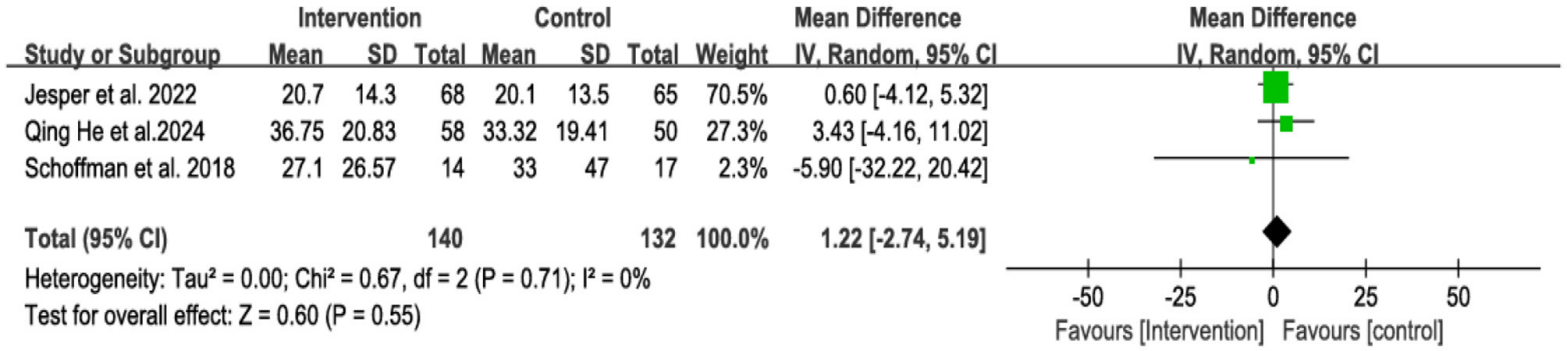
Forest plot of family-centered intervention on MVPA in parents.

Compared with controls, family-centered interventions were not significantly associated with an increase in parental MVPA (mean min/day), with low heterogeneity (WMD = 1.22, 95% CI = −2.74 to 5.19, *p* = 0.55, *I*^2^ = 0%, *p* = 0.71) (Figure 5).

#### 3.4.2 The results of SB

**Figure 6:**
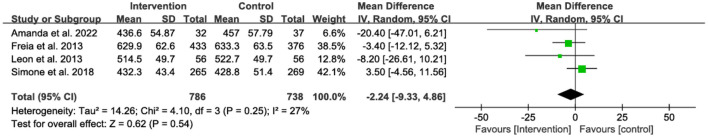
Forest plot of family-centered intervention on SB in children aged under 13.

A meta-analysis of four studies showed that family-centered interventions were not significantly associated with a reduction in children's sedentary time (mean minutes per day) (WMD = −2.24, 95% CI = −9.33 to 4.86, *p* = 0.54). Additionally, no significant heterogeneity was observed (*I*^2^ = 27%; *p* = 0.25) (Figure 6).

## 4 Discussion

### 4.1 Overall effect of family-centered intervention

The aim of this review was to evaluate the effectiveness of family-centered interventions on objectively measured physical activity in children. One may conclude that the family-centered intervention programs were found to be effective in promoting children's MVPA, as evidenced by the 10 selected studies. Notably, significant improvements were observed during weekends and in the short term. However, no significant effect was found on children's sedentary behavior or parental MVPA. An unexpected finding was the lack of change in sedentary behavior despite the increase in MVPA. This result aligns with similar findings from other studies (26). Our findings extended the previous work based on the objective outcomes. Compared with other studies (26, 37), which utilized the mixed outcome measures, the results of this review, which exclusively used objective measurements, are more reliable. Given the lack of previous meta-analyses using a single objective measurement, this review may offer a novel contribution to the field.

Interestingly, social cognitive theory is the most frequently utilized theory in the interventions examined in the included articles. This highlights its potential as a valuable reference for selecting theories in future behavioral interventions. Family interventions should focus not only on the child but also on the parents. Only three articles collected data from the child and the parents. And fundamental movement skills (FMS) should be a novel outcome as early development of FMS is crucial for encouraging PA. Under conditions where equipment permits, gathering data from both parties would provide greater value. However, it is important to note that the included studies used different types of objective devices. Among the ten included studies, only half employed the same devices. The results of this review suggest that high-quality measurement devices were not widely used in earlier studies, although more recent research utilized higher-standard devices. Regarding sample size, only two trials included large-scale samples, with one study involving 534 parent-child dyads and the other 826. While all ten trials were RCTs, two pilot studies had a small sample size. The available data were insufficient to draw definitive conclusions.

### 4.2 Interpretations and suggestions

The findings of this review offer important perspectives on the development of family-focused intervention programs designed to encourage physical activity, especially in children and young adolescents. Firstly, family-centered intervention programs were more effective in the short term. The duration of the intervention appears to be a key influencing factor. This may be because participants initially find the program engaging but gradually lose interest as time progresses. To maintain engagement, future interventions could periodically introduce new themes and incorporate fun, interactive activities to sustain participants' curiosity and enthusiasm. Given that children's interests and levels of engagement vary by age, program design should be developmentally appropriate. Except for a relevant factor of activity duration, additional elements—such as partnerships with external organizations—can further promote participation and extend the program's impact (54). For instance, collaboration with local sports associations, such as youth soccer leagues, can offer structured PA tailored to developmental needs, foster teamwork, and strengthen alignment between program objectives and community resources.

Additionally, the rest period emerged as another potential influencing factor. This review found that family-centered intervention programs had a significant impact on children's MVPA during weekends. During weekdays, children focus on their studies, and parents are often occupied with work, leaving limited time for physical activity. Family-centered interventions should consider maximizing the use of weekdays to encourage physical activity. Besides, leveraging existing resources such as school infrastructure can be an effective strategy for promoting PA (55). One potential approach involves incorporating family-oriented physical activity programs into the school day without increasing academic demands. For instance, schools could implement after-school programs or integrate brief activity breaks during the day that engage both children and their families.

Finally, the role of electronic devices is noteworthy. The review observed no significant effect on reducing sedentary behavior. With the increasing use of electronic devices, children are becoming more sedentary, spending long periods in front of screens. Even physically active children may still engage in excessive sedentary behavior. Family-centered interventions should encourage parents to actively manage screen time by removing or replacing traditional electronic devices at home and promoting outdoor, nature-based activities. Furthermore, it is crucial to acknowledge the increasing interest children have in technology. Future programs could explore integrating electronic devices with PA, such as through active video games or fitness apps that promote movement (56, 57). This approach could harness children's technological interests while fostering physical activity, offering a promising avenue for future interventions.

### 4.3 Strength and limitations

This review has several advantages. Firstly, it is the first one to quantify the effectiveness of family-centered intervention programs which focus on measuring physical activity objectively in children, offering a new perspective on family-centered interventions in this field. While some previous reviews have examined the impact of family-centered physical activity programs, they employed different measurement methods, resulting in significant variability in outcomes. Objective measurement helps reduce bias, further strengthening the findings. Additionally, all included studies were RCTs, which adhere to strict guidelines and ensure high-quality data compared to other experimental designs.

However, this review also has some shortcomings. The sample size was suboptimal, and the objective devices that was used to measure physical activity were very costly, with concerns about losing the devices during the intervention. Given the limited availability of such devices, the sample size should meet the minimum required value, and the latest devices, such as the Actigraph GT3X+, should be considered. Furthermore, most of the included studies were conducted in developed countries, with only one study from a developing country, which may limit the generalizability of the results. Despite these limitations, the review incorporates the most up-to-date and comprehensive research available. Lastly, this study's broad age range of participants may introduce variability in interests, abilities, and motor skills. Such developmental differences could affect the effectiveness of PA interventions, necessitating age-specific adaptations to better address the unique needs of each group. Future studies should consider narrowing the age range and accounting for developmental stages when designing interventions to ensure more tailored and effective outcomes.

## 5 Conclusion

This review evaluated the efficacy of family-centered physical activity interventions for children. The key findings indicate that these interventions positively influence children's MVPA. Further RCTs with longer durations are needed, particularly those targeting reductions in SB and increases in parental MVPA. Well-designed programs should include progressive phases and ensure continuity through external sports activities or programs to foster sustained engagement and long-term behavior change. Family-centered interventions show promise in improving children's MVPA, especially in the short term and on weekends. However, they do not seem to have a significant impact on reducing sedentary time or enhancing parental MVPA. Future research should prioritize strategies to reduce sedentary behavior and focus on high-quality studies with diverse sample populations (e.g., larger-scale samples, populations in developing countries), employing advanced measurement tools, and novel outcomes (e.g., FMS) to more accurately assess the effectiveness of these interventions.

## Data Availability

The original contributions presented in the study are included in the article/Supplementary material, further inquiries can be directed to the corresponding author.
