# Supplementary material for: Effectiveness of family-centered intervention programs on objectively measured physical activity in children aged under 13: a meta-analysis of randomized controlled trials
Source: Front Public Health. 2025 Jul 15;13:1611496. doi: 10.3389/fpubh.2025.1611496 (PMC12303943; doi:10.3389/fpubh.2025.1611496)
Supplement: Supplementary file 1 [file Supplementary_file_1.docx]

| **Search descriptors** |
| --- |
| (("familialities"[All Fields] OR "familiality"[All Fields] OR "familially"[All Fields] OR "familials"[All Fields] OR "familie"[All Fields] OR "family"[MeSH Terms] OR "family"[All Fields] OR "familial"[All Fields] OR "families"[All Fields] OR "family s"[All Fields] OR "familys"[All Fields]) AND ("intervention s"[All Fields] OR "interventions"[All Fields] OR "interventive"[All Fields] OR "methods"[MeSH Terms] OR "methods"[All Fields] OR "intervention"[All Fields] OR "interventional"[All Fields]) AND ("exercise"[MeSH Terms] OR "exercise"[All Fields] OR ("physical"[All Fields] AND "activity"[All Fields]) OR "physical activity"[All Fields]) AND ("child"[MeSH Terms] OR "child"[All Fields] OR "children"[All Fields] OR "child s"[All Fields] OR "children s"[All Fields] OR "childrens"[All Fields] OR "childs"[All Fields]) AND ("parent s"[All Fields] OR "parentally"[All Fields] OR "parentals"[All Fields] OR "parented"[All Fields] OR "parenting"[MeSH Terms] OR "parenting"[All Fields] OR "parents"[MeSH Terms] OR "parents"[All Fields] OR "parent"[All Fields] OR "parental"[All Fields])) |
